# Heterointerface Engineering of β-Chitin/Carbon Nano-Onions/Ni–P Composites with Boosted Maxwell-Wagner-Sillars Effect for Highly Efficient Electromagnetic Wave Response and Thermal Management

**DOI:** 10.1007/s40820-022-00804-w

**Published:** 2022-03-29

**Authors:** Fei Pan, Lei Cai, Yuyang Shi, Yanyan Dong, Xiaojie Zhu, Jie Cheng, Haojie Jiang, Xiao Wang, Yifeng Jiang, Wei Lu

**Affiliations:** 1 Shanghai Key Laboratory of D&A for Metal-Functional Materials, School of Materials Science & Engineering, Tongji University, Shanghai, 201804 People’s Republic of China; 2 School of Materials Science and Engineering, University of New South Wales, Sydney, 2052 Australia

**Keywords:** β-chitin, Nano onion carbon, Electromagnetic wave absorption, Electromagnetic interference shielding, Photothermal

## Abstract

The rational construction of microstructure and composition with enhanced Maxwell-Wagner-Sillars effect (MWSE) is still a challenging direction for reinforcing electromagnetic wave (EMW) absorption performance, and the related EMW attenuation mechanism has rarely been elucidated. Herein, MWSE boosted β-chitin/carbon nano-onions/Ni–P composites is prepared according to the heterointerface engineering strategy via facile layer-by-layer electrostatic assembly and electroless plating techniques. The heterogeneous interface is reinforced from the aspect of porous skeleton, nanomaterials and multilayer construction. The composites exhibit competitive EMW response mechanism between the conductive loss and the polarization/magnetic loss, as describing like the story of “The Hare and the Tortoise”. As a result, the composites not only achieve a minimum reflection loss (RL_min_) of − 50.83 dB and an effective bandwidth of 6.8 GHz, but also present remarkable EMW interference shielding effectiveness of 66.66 dB. In addition, diverse functions such as good thermal insulation, infrared shielding and photothermal performance were also achieved in the hybrid composites as a result of intrinsic morphology and chemicophysics properties. Therefore, we believe that the boosted MWSE open up a novel orientation toward developing multifunctional composites with high-efficient EMW response and thermal management.

**Supplementary Information:**

The online version contains supplementary material available at 10.1007/s40820-022-00804-w.

## Highlights

- The squid pen-derived aerogel with intrinsic electropositivity is prepared to electrostatically assemble carbon nano-onion with low escape energy.
- Under the guidance of PNM model, the interface polarization is reinforced from the aspect of porous skeleton, nanomaterials and multilayer construction.
- Benefiting from boosted Maxwell-Wagner-Sillars effect, the aerogel and film display remarkable electromagnetic wave absorption (−50.83 dB) and electromagnetic interference shielding performance (66.66 dB), respectively.

**Supplementary Information:**

The online version contains supplementary material available at 10.1007/s40820-022-00804-w.

## Graphical abstract

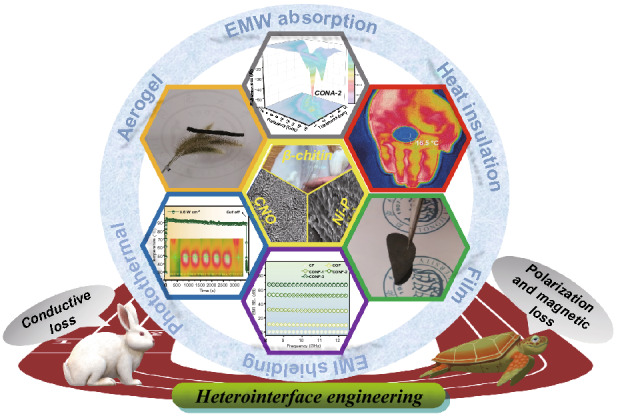

## Introduction

In principle, multicomponent electromagnetic wave (EMW) absorber can endow materials with adequate heterogeneous interface and improved impedance matching, facilitating EMW response and dissipation to cope with the unwanted electromagnetic pollution problem in current 5G life [1, 2]. Maxwell-Wagner-Sillars effect (MWSE), is generated in the heterojunction structures because diverse charge distribution at the boundary between two regions with different dielectric properties [3, 4]. Simultaneously, the capacitor-like interfaces will accordingly form a local dipole electric field, and thus, assembled movements of interfacial dipoles intensify the responsive process with input EMW field, which boost the attenuation of EMW [5]. In view of this prerequisites, fabricating multicomponent composites with abundant heterogeneous interface becomes a mainstream strategy for enhancing MWSE, as well as obtaining highly efficient EMW response materials [6]. Hitherto, diverse adoptable routes have been established to optimize MWSE in multicomponent composites via heterogeneous interface engineering and rational construction. For all this, composites with maximized heterogeneous interface area is still regarded as a challenging direction for reinforcing EMW response, thereby putting forward brand-new requirements of the morphology and size within single-component as well as combination between multi-components [7, 8].

To overcome this challenge, two pivotal points should be grasped into consideration: (i) how to increase the loading area of the basal layer, and (ii) how to increase the contacting area between basal layer and composite layer. According to the aforementioned considerations, a PNM model based on porous skeleton, nanomaterials and multilayer construction can provide a feasible approach, as shown in Fig. S1. It is unambiguous that the surface area of porous materials is higher than that of the solid counterparts at the same volume [9, 10]. In addition, on the basis of Maxwell-Garnett theory, the porous structure is conductive to manipulating the permittivity of the system due to the introduction of air medium as well, thus reinforcing impedance matching degree and internal multiple scattering. Owing to the characteristics of lightweight, high porosity and specific surface area, aerogel is regarded as an idea basal layer for loading composite unit. In recent years, a variety of aerogels based on MXene, graphene, gelatin, bacterial cellulose, and so on have been successfully fabricated [11–13]. As the only positively charged basic polysaccharide in nature, β-chitin, can adhesion other dielectric loss type materials with negatively charged via electrostatic self-assemble, thereby dramatically increasing the MWSE in comparison with the aforementioned aerogel materials. This assembles method gives full play to intrinsic charge characteristics of the material itself, which greatly avoids the treatment of surfactant or complicated process during conventional composite strategies. Besides, the parallel molecular chains interior the β-chitin reduce the intermolecular hydrogen bond force, which makes the β-chitin derived aerogel process have better chemical activity and absorbability as a skeleton. Unfortunately, few works about β-chitin derived aerogel were reported yet, making it extremely indispensable to utilize the β-chitin derived aerogel as the basal layer.

For the composite layer, due to their superior specific surface area, nanomaterials are good choices, thus leading to the explosion of contract area between basal layer, as well as the promoted MWSE. The contacting area per unit increases with the decrease of the size. Moreover, since the particle size is at the nanoscale, the nanomaterials also generate expanded dipole polarization and electron polarization through the increased defect, dipoles and dangling bonds [14, 15]. As another attractive member of the fullerene family after C60, carbon nanotubes and graphene, carbon nano-onion (CNO) is composed of concentric graphite shells with an ultra-small size of about 5 nm. The CNO particles possess unique characteristic of large specific surface area, excellent electrical conductivity, stable thermal and chemical stability, endowing it great application potential in the field of electromagnetic functional materials [16, 17]. At the same time, the hollow structure among the graphite shell also contributes to the multiple reflections of incident EMW in the cavities [18]. In addition, as a low escape energy material similar to carbon nanotube, the electric field formed around CNO can easily cause electrons to emit. When CNO particles with negative charge approach to β-chitin with positive charge, it can be foreseen that a tightly connected interface will be generated under the electrostatic force, and the whole surface area of the porous structure with the assistance of nanostructure can be effectively utilize. In view of these merits aforementioned, the combination of CNO with β-chitin aerogel makes a good use of the core strategy that high contacting area promotes interface polarization. Besides, the introduction of CNO into aerogels also build a 3D conductive loss network and improve the migration of charges on unpyrolyzed β-chitin.

Apart from the nanomaterial regulation, another underlying issue that we are considered to further increase the contacting area between heterogeneous interfaces is multilayer construction. Hamburger, the most typical layered structure in daily, is stood out as the basic design framework for the construction of multilayer composites, which can be simply divided into five layers. Magnetic nanoparticles have been selected as the most suitable outermost layer among the total multi-materials. It can not only further increase the MWSE for the formation of heterogeneous interfaces with the middle layer, but also enrich the mechanism of electromagnetic loss via the conception of electromagnetic coupling. As a kind of magnetic loss type material with internal long range disordered arrangement, amorphous magnetic alloy has been neglected in the field of EMW absorption for its low permeability compared with crystalline magnetic substitute [19]. However, when the amorphous magnetic alloy is in contact with the air as its outermost layer, the impedance matching can be preferably optimized due to the moderate conductivity of the amorphous material, and it could reduce the reflection of the EMW when it contacts with the outermost layer. Among the methods for preparing amorphous magnetic alloys, because of its facile equipment, low pollution and low cost, electroless plating is widely spread and their product Ni–P has attracted extensive attention on account of its properties of good uniformity, compact growth and corrosion resistance. Under the assistant of facial equipment, electroless plating can be used to obtain magnetic layers on the surface of carbon materials with various shapes. The high-density amorphous coating on the surface with no trace of fracture could build the second pathway for charge migration. Additionally, the intrinsic ferromagnetic characteristics also introduce magnetic loss into the system, thus enriching the loss mechanism to some extent [20]. Therefore, it is evidently attempted to employ electroless plating for deeply elevating MWSE.

Inspired by above, a β-chitin/carbon nano-onions/Ni–P aerogel (CONA) based on PNM model was prepared in this work for the first time. Through layer-by-layer electrostatic assembly, samples with five layers were attained to boost the MWSE of composites and simultaneously regulate other loss mechanisms of EMW responses. In detail, under the guidance of PNM model, the porous skeleton, nanomaterials and multilayer construction maximizes the loading area and contact area of the composites. Ultra-tiny CNO and amorphous Ni–P was firstly served as the middle layer and outer layer, which help building 3D conductive loss and magnetic loss network, respectively. This microscopic manipulation and appropriate multicomponent design endow CONA with excellent thermal insulation and EMW absorption capacity, where the RL_min_ of − 50.83 dB and effective bandwidth of 6.8 GHz was obtained. By adjusting the rounds of electroless plating, the conductive loss and polarization/magnetic loss of CONA exhibit opposite trends, as describing like the story of “The Hare and the Tortoise”. Moreover, a flexible β-chitin/carbon nano-onions/Ni–P film (CONF) was also fabricated via similar method and shows outstanding electromagnetic interference (EMI) shielding (66.66 dB) and photothermal integrally. Overall, this work put forward an interesting construction in developing highly efficient multifunctional EMW response materials with boosted MWSE from aspect of increasing the heterogeneous interface area, providing a guiding pathway for the follow-up works.

## Experimental

### Materials

All chemicals including stannous chloride (SnCl_2_), lead chloride (PbCl_2_), sodium hypophosphite (NaH_2_PO_2_·2H_2_O), nickel (II) sulfate hexahydrate (NiSO_4_·6H_2_O), trisodium citrate (Na_3_C_6_H_5_O_7_·2H_2_O), ammonium chloride (NH_4_Cl), ammonium hydroxide (NH_4_OH) sodium hydroxide (NaOH), hydrochloric acid (HCl), and acetic acid (CH_3_COOH) were purchased from Sinopharm Chemical Reagent Co., Ltd., Beijing, China. Squid pen was purchased from supermarket nearby. CNO was purchased from Nanjing Mingchang New Material Co. All chemical reagents used as received without further purification.

### Preparation of β-Chitin Dispersions

The squid pen was firstly washed with deionized water and clipped into small slices of 0.5 × 1 cm^2^. 5 g squid pen was immersed in 1 mol L^−1^ HCl aqueous solution for 1 h with continuous stirring to remove minerals. Then, the obtained produce was dispersed in 500 mL of aqueous solution containing 20% ethanol and stirred for 12 h to exclude the lipids. After washing with deionized water until the pH is neutral, products were dispersed to 2.5 M NaOH solution and stirred for another 12 h to exclude the protein, obtaining the primary β-chitin. After washing with same washing process, the above primary β-chitin go through a protonated process, where product was immersed into 4% acetic acid solution and stirred for 12 h. Finally, a wet grinder was employed to grind the primary β-chitin twice to fabricate refined β-chitin dispersions.

### Preparation of COA

The pure β-chitin aerogel (CA) was prepared in a freeze drying, where 10 mL β-chitin dispersions (5 mg mL^−1^) was placed in the refrigerator for 12 h and then freeze-dried at − 80 °C for 2 days. For preparing β-chitin/CNO aerogel (COA), the obtained CA were further coated by CNO through an electrostatic adhesion approach. Typically, CA was immersed into CNO solution (500 mg CNO and 100 mL deionized water) and placed in a shaker for 1 day. At last, the wet β-chitin/CNO composite was placed in the refrigerator for 12 h and then freeze-dried at − 80 °C for 2 days, obtaining COA.

### Preparation of CONA

The CONA were synthesized via electroless plating method, and the preparation process is similar to COA except for the final freeze drying. The wet β-chitin/CNO composite was firstly was dispersed into a mixed solution (SnCl_2_ 2 g; H_2_SO_4_ 10 mL; deionized water 90 mL) with ultrasonic vibration for 15 min. Then, the treated product was dispersed into a mixed solution (PbCl_2_ 0.025 g; HCl 0.25 mL; deionized water 99.75 mL) with ultrasonic vibration for another 15 min. Afterward, the treated product immersed in mixed solution (NaH_2_PO_2_·2H_2_O 1 g; deionized water 100 mL) with ultrasonic vibration for 3 min. Finally, the above-treated wet β-chitin/CNO composite were subsequently put into 150 mL nickel electroless plating solution at 60 °C for 20 min. Herein, electroless plating solution was mixed with of 6 g NiSO_4_·6H_2_O, 3 g NaH_2_PO_2_·2H_2_O, 15 g Na_3_C_6_H_5_O_7_·2H_2_O and 7.5 g NH_4_Cl. The pH value is adjusted to 9 via NH_4_OH. The electroless plating process is repeated one to three times, and the resulting CONA is named as CONA-1, CONA-2, and CONA-3, respectively.

### Preparation of CONF

The CONF were synthesized via hot press process and following electroless plating method. Typically, 2.5 mL β-Chitin dispersions (5 mg mL^−1^) and 50 mg CNO are stirred and translate to the filtration device to get wet β-Chitin/CNO film (COF). Then, the wet COF is placed in a Teflon model for hot pressing in place, and COF is obtained by drying at 60 °C for 12 h. Afterward, the COF undergo the same electroless plating process as CONA. The electroless plating process is repeated one to three times, and the resulting CONF is named as CONF-1, CONF-2, and CONF-3, respectively.

### Characterization

The chemical composition of the samples was characterized via X-ray diffractometer (XRD, DX-2700) and use Cu-Ka radiation (*λ* = 1.54 Å). The morphology and microstructure were observed with scanning electron microscopy (SEM) and transmission electron microscopy (TEM). Fourier transform infrared (FTIR) spectra of the samples were investigated by FTIR spectrophotometer (The Nicolet iN10). The surface composition and valence state of elements were measured by X-ray photoelectron spectroscopy (XPS, Thermo Scientific K-Alpha). A standard four-probe station (HPS2524) was investigated for measuring the electrical conductivity of the samples. The photothermal performance test was measured under an 808 nm NIR laser system with power densities from 0.1 to 0.9 W cm^−2^, and the corresponded thermal images were captured by infrared thermal imaging device (FLIR ONE PRO). The EMW parameters measured by a vector network analyzer (VNA, 3672B-S, Ceyear). The samples were mixed in paraffin matrix with 35 wt%, which were shaped into a circular ring with an internal diameter of 3.0 mm and external diameter of 7.0 mm. The EMI shielding performance of the samples were tested by a vector network analyzer (VNA, 3672B-S, Ceyear) utilizing the wavelength over the entire frequency range of the X-band (8.2−12.4 GHz), and the specific formula is described as supplementary material. Radar Cross-Section (RCS) simulation was studied in CST software and described in the supporting information detailly.

## Results and Discussion

### Fabrication and Characterization of CONA and CONF

**Fig. 1:**
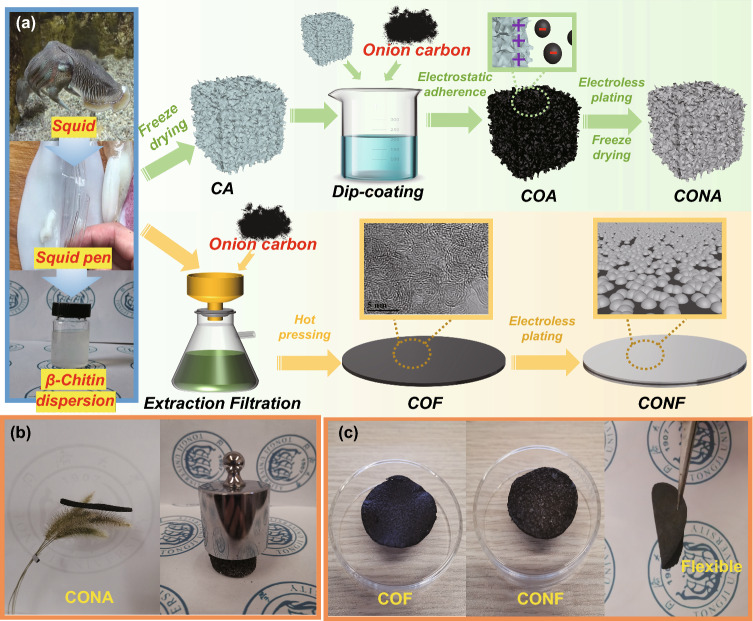
**a** Schematic illustrating the synthetic processes of CONA/CONF; **b** digital images exhibit the CONA standing on dandelion and compression resistance performance; **c** digital images display flexible COF and CONF

The synthetic processes for five-layered CONA are shown in Fig. 1a. In the initial step, the natural squid pen was regularly cut into flakelets and immerse into HCl, ethyl alcohol, NaOH and CH-COOH for removing minerals, oils and proteins, respectively. After following wet grinding and freeze-drying treatment, the obtained CA with positive charge electrostatic adhered CNO with negative charge, and formed COA that process strong coupling interface (detail electrostatic adhesion is proved in Fig. S2). Finally, CONA was fabricated via conventional electroless plating. From the digital image of Fig. 1b, the porous structure and excellent binding force of CA skeleton endowed CONA with lightweight and compression resistance ability. The as-prepared CONA can stand on setaria viridis and tolerate 400 times pressure with its own weight. Similarly, CONF was prepared by using β-chitin dispersion as mechanical enhancing phase and CNO and Ni–P as electromagnetic response layer. After filtration, hot pressing, and subsequent electroless plating process, the black COF surface developed a silver sheen and the resulting CONF exhibits flexible characteristic, as displayed in Fig. 1c.

**Fig. 2:**
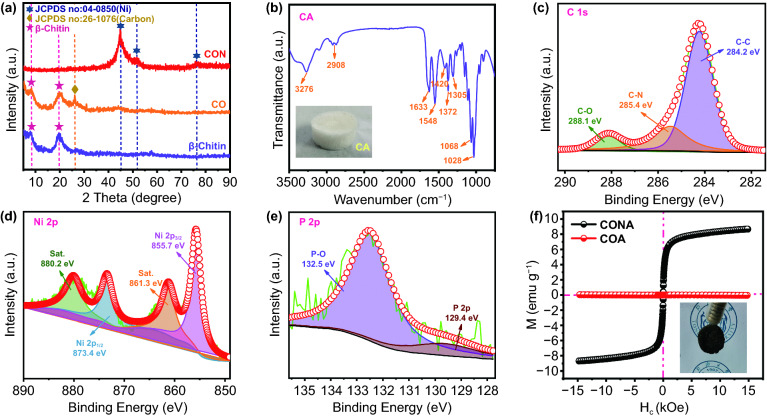
**a** XRD patterns of samples; **b** FTIR spectra of CA; high-resolution XPS survey: **c** C 1s, **d** Ni 2p, and **e** Ni 2p; **f** room-temperature hysteresis loops of COA and CONA

XRD is employed to reveal the crystallographic structures of the as-obtained composites (Fig. 2a). Typical characteristic peaks appeared in 8° and 20° in pure β-chitin and β-chitin/CNO samples, confirming the existence of β-chitin. Besides, additional diffraction peak in near 27° is coincided with the (006) planes of carbon (JCPDS No. 26–1076). After the electroless plating step, β-chitin/carbon nano-onions/Ni–P composites displayed obvious amorphous and nanocrystalline peaks at 44°, 51°, and 76°, which were assigned to (111), (200), and (220) planes of Ni, respectively (JCPDS 04–0850) [21]. The chemical composition of β-chitin is further detected by the FTIR, as shown in Fig. 2b. CA sample shows characteristic peaks of amide I at 1633 cm^−1^, amide II at 1548 cm^−1^, and C–H at 1372 cm^−1^, which can be ascribed to the β-chitin in accordance with previous report [22]. In particular, β-chitin has a coaligned molecular chain structure resulting in low hydrogen bond interaction. Thus, single characteristic peak at 1633 cm^−1^ is distinguished from bimodal state of α-chitin. The elemental chemical states of CONA-2 are characterized by XPS measurements (Figs. 2c–e and S3). The high-resolution C 1s spectrum (Fig. 4b) showed CONA with a binding energy at 288.1, 285.4, and 284.2 eV, assigning to the C–O, C–N, and C–C bands, respectively [23]. The Ni 1s spectra of the CONA is fitted into four main peaks at 880.2, 873.4, 861.3, and 855.7 eV, which are corresponded to the satellite peaks I, Ni 2p_1/2_, satellite peaks II and Ni 2p_3/2_, the accompanied satellite peaks, respectively [24]. In addition, two peaks at 132.5 and 129.4 eV are connected with the phosphate species and P^δ−^ 2p_1/2_ of phosphides, respectively [23]. This result, along with the XRD and FTIR analysis, illustrate that the ternary component of β-chitin/carbon nano-onions/Ni–P is successfully fabricated. According to the result of magnetic hysteresis loops (Fig. 2f), the CONA possesses a higher saturation magnetization than COA as a result of the intrinsic ferromagnetic characteristics Ni–P, which is benefited to promote the initial permeability and hence the magnetic loss properties [23].

**Fig. 3:**
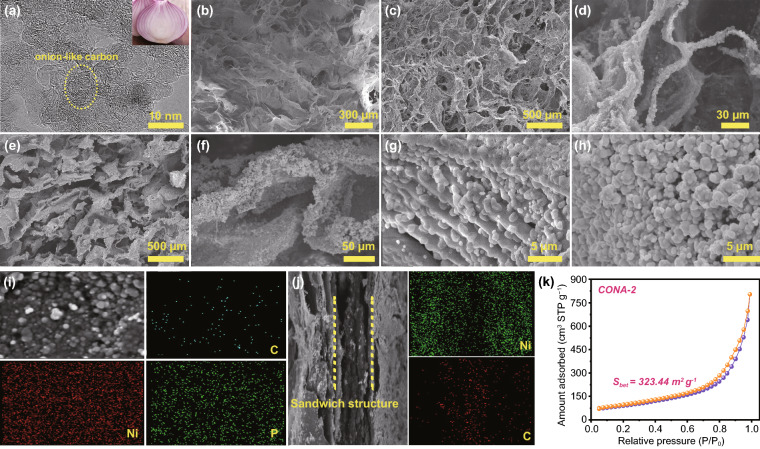
**a** TEM images of CNO; SEM images of **b** CA, **c-d** COA, and **e–h** CONA-2; EDS of C, Ni, and P elements of CONA-2 **i** and EDS of C and Ni elements of CONF-2 **j**; **k** N_2_ adsorption/desorption isotherms of CONA-2

The microstructures of the obtained CONA and CONF are characterized by TEM and SEM (Fig. 3 and Fig. S4, S5). As elucidated from the high-resolution TEM image in Figs. 3a and S4, the distinct lattice fringe clearly demonstrates that each CNO, with the size of about ~ 5 nm, possesses an onion-like structure that consist of several concentric graphite shells. TEM images also unambiguously display that plenty of CNO with uniform size are piled and formed in each region, thereby increasing the specific surface area of the composite layer to a certain extent. For CA, due to the sublimation of ice crystals during the freeze-drying process, Fig. 3b exhibits the interlinked three-dimensional porous structure. After electrostatic adhesion with CNO, the cross-sections of COA exhibit a rough porous structure with countless nanoparticles covering on the β-chitin, and the holes in the longitudinal plane became expansion (Fig. 3c–d). Figure 3e–f reveal the images of CONA-2, where Ni–P layer exhibit a coarser and denser distribution on the surface of CA after electroless plating process. As shown in Fig. 3g, h, the amorphous Ni–P is a spherical structure with a diameter of about 2 μm. Meanwhile, the sufficient contact between aerogel and solution during electroless plating facilitates the uniform distribution of Ni–P particles on the surface and gap of porous structure, which indicates that the two layers are well combined. Besides, the densification of Ni–P particles increased with the increasing electroless plating rounds. Figure S5e-f display the SEM images of the CONF-2, confirming that the CNO is tightly wrapped by β-chitin, accompanied by granular Ni–P on the surface. Moreover, according to the cross-sectional image, the thickness is about 82 µm. To investigate the distributions of elements in the CONA-2 and CONF-2, elemental EDS mapping of carbon (C), nickel (Ni), and phosphorus (P) are further detected. As shown in Fig. 3i the C, Ni, and P elements are uniformly distributed over the β-chitin skeleton in CONA-2. And an obvious sandwich element distribution can be observed in CONF-2, where Ni intersperses on both sides of C (Fig. 3j). The nitrogen adsorption/desorption curve is employed to detect the specific surface areas of porous samples. As shown in Fig. 3k, the curves of CONA-2 display an IV-type isotherm and the calculated specific surface areas 323.44 m^2^g^−1^.

### EMW Absorption Performance of CONA

**Fig. 4:**
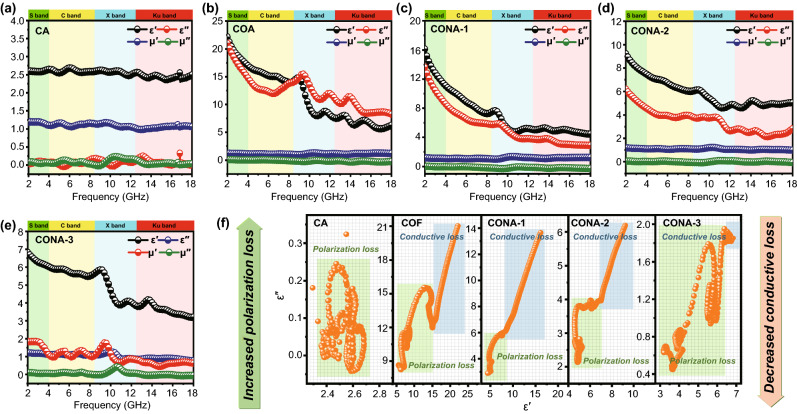
EMW parameters of **a** CA; **b** COA; **c** CONA-1; **d** CONA-2; **e** CONA-3. **f** Cole–Cole semicircle of samples

The tendency of the complex permittivity (*ε*_r_ = *ε*′ − j*ε*″) and complex permeability (μr = μ′ − jμ″) with frequency from 2 to 18 GHz is investigated for the EMW absorbing properties of the as-prepared samples. It is well-known that the real parts and imaginary parts of EMW parameters stand for the storage and the attenuation ability of EMW energy, respectively [25, 26]. It is observed from Fig. 4 that the ε′ values of samples display a decreasing tendency in the frequency range of 2 to 18 GHz except CA, which is mainly caused by the polarization hysteresis phenomenon under reaction of high-frequency electric field. Due to intrinsic insulating properties, CA has the lowest ε′ and ε″ values, which represents poorest storage and dissipation capacity with inside EMW. Considering the introduction of the CNO with highly conductivity, it is reasonable that the COA exhibits boosted permittivity due to the reinforcement effect of dielectric loss, whose ε′ and ε″ values vary from 22.14 and 21.02 at 2.0 GHz to 6.25 and 8.23 at 18.0 GHz, respectively. However, absorber with excessive permittivity will cause reflection of contacted EMW on the surface instead of accessing to the interior, thereby impeding subsequent EMW response. After electroless plating, as exhibited in Fig. 4c–e, CONA-1, CONA-2, and CONA-3 exhibit optimized and tunable permittivity in the frequency range of 2.0–18.0 GHz. With the increased rounds of electroless plating, both the ε′ and ε″ values show a decreasing dependence, implying that the conductivity of amorphous Ni–P is lower than that of CNO. For example, CONA-1 presents the highest ε′ and ε″ values that gradually decrease from 16.12 and 13.62 at 2.0 GHz to 4.45 and 2.88 at 18.0 GHz, respectively. When more Ni–P particles grow on the surface of CNO, ε′ values of CONA-2 and CONA-3 are prospectively varied from 9.22 to 5.04 and from 6.82 to 3.20, respectively, and the corresponding ε″ values are decreased from 6.19 to 2.82 and from 1.85 to 0.63, respectively. Based on acknowledged dielectric theory, the dielectric loss is mainly determined by conductive loss and polarization loss [27, 28]. The former generally generates from the energy transformation from electrical energy formed by charge migration to heat energy in dielectric loss medium, and later usually comes into being from MWSE or dipole polarization when the polarization of the dielectric lags behind the applied EM field. The conductivity is proportional to ε″ according to the free-electron theory (ε″ = σ/2πfε_0_) [29]. Herein, one can find that the conductivity of the samples is in the order of COA > CONA-1 > CONA-2 > CONA-3 > CA, that is, their intrinsic conductive loss is in the same order. The coating of CNO layer over CA achieve electric transportation throughout the 2D β-chitin laminas by migrating in conductive channels, and a 3D conductive network is further built via CA as a skeleton. The addition of Ni–P particles with relatively low conductivity disrupts the balance of the current network, thus forcing charges to consume more energy to overcome these “barriers”. Therefore, the samples’ ability to convert EM energy into heat through conductive loss is significantly reduced, and this phenomenon becomes more significant as the number of absorbed particles increases. Besides, polarization loss is another crucial factor that is expressed by Cole–Cole semicircle model [30, 31]:

1 $$\left( {\varepsilon^{\prime} - \frac{{\varepsilon_{s} + \varepsilon_{\infty } }}{2}} \right) + \left( {\varepsilon^{\prime\prime}} \right)^{2} = \left( {\frac{{\varepsilon_{s} - \varepsilon_{\infty } }}{2}} \right)^{2}$$

and the *ε*′ and *ε*″ can be deduced into:

2 $$\varepsilon^{\prime} = \varepsilon_{\infty } + \frac{{\varepsilon_{s} - \varepsilon_{\infty } }}{{1 + \left( {2\pi f\tau } \right)^{2} }}$$

3 $$\varepsilon^{\prime\prime} = \varepsilon_{\infty } + \frac{{2\pi f\tau (\varepsilon_{s} - \varepsilon_{\infty } )}}{{1 + \left( {2\pi f\tau } \right)^{2} }}$$

$\varepsilon_{s}$, $\varepsilon_{\infty }$, and $\tau$ stand for the static dielectric constant, the dielectric constant at infinite frequency and the polarization relaxation time, respectively. In the *ε*’-*ε*’’ curves, the leftward standard semicircles area represents the polarization loss and the “tail” at the rightward area suggests conductive loss on the basis of Liu’s work [32].The strength of the Debye dipolar relaxation and conductive loss is determined by the number/radius of semicircles, and the length of the “tail”, respectively [33]. When taking a close look in Fig. 4f, it is obvious to find out that the numbers of semicircles surge from CA to COA and from COA to CONA-1, further increasing as the electroless plating rounds intensify. Boosted Debye dipolar relaxation is a sign that MWSE is acting a dominant role in polarization loss, which can be explained by as-mentioned PNM model. In the heterostructures, the difference of electronegativity between two materials will generate a capacitor-like structure, forming built-in electric fields and space charge regions, which influences charge transport and movements of interfacial dipoles [34]. When the assembled interfacial dipoles amplify the response to alternate EM field and thus contribute to EMW absorbing ability. The “tail” gradient on the ε’-ε’’ curves of samples firstly increase and then decreases with the increased round number of electroless plating. The absence of the tail for CA and the small slope of CONA-3 indicate the poor conductivity, being in keeping with the previous rule of conductive loss.

**Fig. 5:**
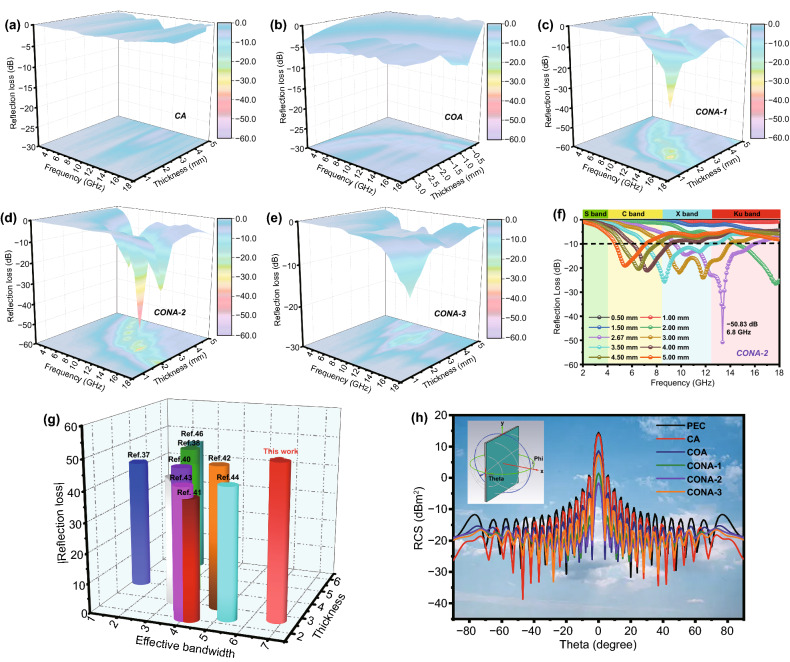
3D RL performance of **a** CA, **b** COA, **c** CONA-1, **d** CONA-2 and **e** CONA-3 in 2–18 GHz range from 0.5–5.0 mm; **f** 2D RL performance of CONA-2; **g** EMW absorption performance in comparison of reported aerogel absorbers; **h** RCS simulated image of PEC contained composites with scanning angles from -90° to 90°

Figure 5a–f display the EMW absorption performance of aerogel samples in the frequency range of 2.0–18.0 GHz, which was depicted by following formula [35]:

4 $$Z_{in} = Z_{0} \left( {\mu_{r} \varepsilon_{r} } \right)^{1/2} \tanh \left[ {j(2\pi fd\left( {\mu_{r} \varepsilon_{r} } \right)^{1/2} /c} \right]$$

5 $${\text{RL}}\left( {{\text{dB}}} \right) = 20\log \left| {\left( {Z_{in} - Z_{0} } \right)/\left( {Z_{in} + Z_{0} } \right)} \right|$$

Among them, *Z*_0_, *Z*_in_, *d* and *c* refer to the impedance of free space, impedance of the absorber, absorber thickness, and velocity of light, respectively. Besides, RL <  − 10 dB suggests that the absorber can attenuate 90% EMW, and the corresponding frequency range is called effective absorption bandwidth (EAB). From Fig. 5a–b, as for the CA and COA, from the intuitive information, it can be observed that RL at all thickness are higher than −10 dB due to its insulation characteristics and impedance mismatch, respectively, which indicates the poor EMW response ability. Due to the uniform dispersion of magnetic Ni–P particles during electroless process, CONA-1, CONA-2, and CONA-3 display the improved RL and EAB value. The multilayer CONA-1 sample exhibits a RL_min_ value of − 35.8 dB at 14.0 GHz at only a 2.30 mm thickness (Fig. 5c). And at the same thickness of 2.30 mm, the EAB of CONA-1 reaches up to 7.1 GHz (10.9–18.0 GHz), which reveals the ultrawide EMW absorption performance. For the CONA-2 composite with growing amorphous Ni–P shell, its bottommost RL peak at 9.0 GHz climb to − 50.8 dB at the thickness of 2.67 mm and the corresponded EAB reach 6.6 GHz (9.4–16.0 GHz), suggesting superior EMW absorption capability (Fig. 5d). When further increasing the round of the electroless plating, the final EMW performance of CONA-3 present a downward trend, where the widest EAB of 2.4 GHz and the RL_min_ value of −19.3 dB at 3.5 mm are achieved (Fig. 5e). Among the Ni–P coated absorbers, it can be concluded that CONA-2 exhibits the lowest RL_min_ and the EAB covers 83% of the measuring frequency when the matching thickness is tuned from 0.5 to 5.0 mm (Fig. 5f). Furthermore, the frequency corresponding to the lowest RL peak moves to the low frequencies with the increased of thickness, which can be well in accordance with the quarter-wavelength model (Fig. S8). When the 180° phase difference between the incident and reflected EMW in the absorber generates, the induced interference leads to the total offset of the reflected EMW at the absorber–air interface [32]. The RL_min_, EAB and matching thickness characteristics of representative aerogel materials with similar porous structure reported in recent literature are shown in Fig. 5g to comprehensively assess the EMW absorption performance of CONA­2 (detail in Table S1) [36–45]. It is noteworthy that CONA­2 assuredly achieve powerful RL and broad EAB in thin matching thickness, exhibiting prominent advantages to those reported EMW absorbers. Furthermore, the RCS simulation is adopted to evaluate EMW absorption performance of samples under actual application situations by the CST microwave software [46]. The double layered simulation model is composed of the as-prepared samples layer with 2.67 mm and perfect electric conductor (PEC) layer with 5.00 mm (Fig. S9). For Fig. 5h, the RCS distribution on the Y − O − Z surface between -90°-90° is illustrated and the corresponding RCS reduction is achieved by subtracting the samples layer with the PEC layer. In addition to the CA, the vertical reflected intensity of the samples is weaker than that of pure PEC, implying that EMW signal can be attenuated by the coating absorbing layer. In particular, it can be demonstrated that the CONA-2 displays the highest RCS reduction value (16.06 dB m^2^) at 0° than that of other aerogels, which is in accordance with the EMW absorption results that are deduced by transmission line theory. In other words, when the substrate plane is coated with the CONA-2, the incident EMW energy will profitably dissipate.

**Fig. 6:**
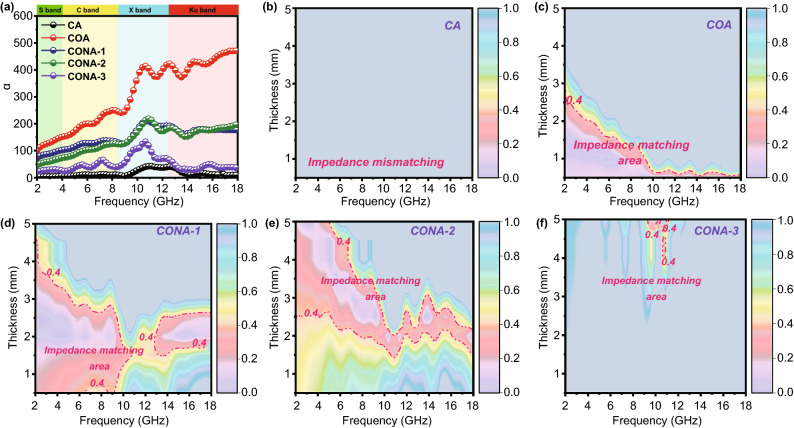
**a** Attenuation constant of samples; calculated $\vartriangle$ value maps: **b** CA, **c** COA, **d** CONA-1, **e** CONA-2 and **f** CONA-3

To appraise the internal EMW absorption capacity of samples, two conditions, including attenuation constant (*α*) and impedance matching, are clearly illustrated in Fig. 6. *α* is related to the EMW energy assimilated by the absorber and impedance matching is associated with the degree that EMW propagates the inside of the absorber, which are evaluated by the following formulas [47, 48]:

6 $${\upalpha } = { }\frac{\sqrt 2 }{c}\pi f\sqrt {\left( {\varepsilon^{\prime\prime}\mu^{\prime\prime} - \varepsilon^{\prime}\mu^{\prime}} \right) + \sqrt {\left( {\varepsilon^{\prime\prime}\mu^{\prime\prime} - \varepsilon^{\prime}\mu^{\prime}} \right)^{2} - \left( {\varepsilon^{\prime\prime}\mu^{\prime} + \varepsilon^{\prime}\mu^{\prime\prime}} \right)^{2} } }$$

7 $$\left| \vartriangle \right| = \left| {\sinh^{2} \left( {{\text{Kfd}}} \right) - M} \right|$$

where *M* and *K* parameter are calculated by the EM parameters and detailly descripted in Supporting Information (formula S1). The large area with delta value ($\vartriangle$) between 0 and 0.4 represents a satisfactory degree of impedance matching [49, 50]. By calculation, it is obvious that α of tested aerogels is in the order of COA > CONA-1 > CONA-2 > CONA-3 > CA (Fig. 6a) that follows the same rule of *ε*″, indicating that elevated conductive loss is beneficial to the attenuation of EMW energy. However, owing to the superfluous reflection of EMW in high conductivity material, seriously impedance mismatching happens and lead to a poor EMW absorption property. The $\vartriangle$ values of samples at different thicknesses are calculated and showed in Fig. 6b–f. As a comparison, the impedance matching area of the CA and CONA-3 is almost negligible, and most of them are higher than 1 in the entire frequency range, revealing the impedance mismatching condition. On the contrary, CONA-1 and CONA-2 display enlarged area where more $\vartriangle$ values close to zero, indicating an appropriate impedance matching condition. It is supposed that MWSE engineering with hierarchically coating lower conductive layer harmonizes the huge impedance gap between the CNO and air, leading to smooth channel for EMW transmission internally. All these inferences mentioned above certify that the CONA-1 and CONA-2 with integrated attenuation capability and impedance matching are considered as significant factors for our samples as an ideal EMW absorber.

**Fig. 7:**
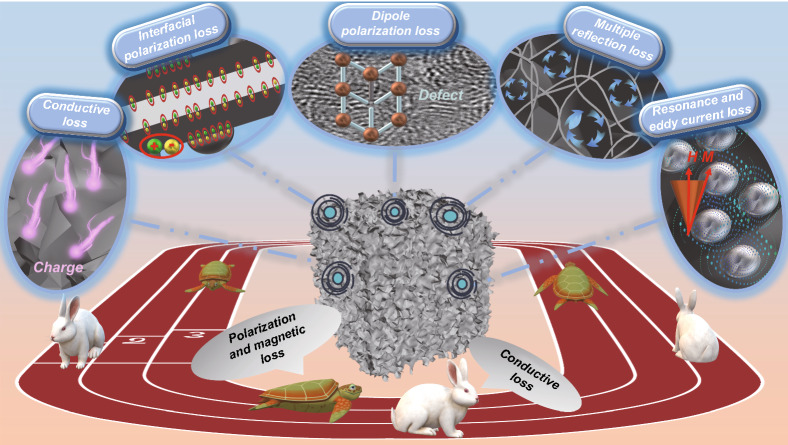
Possible EMW absorption mechanisms for CONA-2

According to the previous elaboration of the EMW parameters and the EMW absorption performance, Fig. 7 illustrates the cogitative EMW absorption mechanism, which includes the conductive loss, MWSE, dipole polarization, multiple reflection loss and magnetic loss. Firstly, as one of zero-dimensional carbon cluster, conductive CNO act as a dominant part in the generation of the conductive network in the 3D β-chitin aerogel skeleton, where EMW energy is converted into heat energy and consumed [51]. Second and most important, boosted MWSE derives from heterogeneous interface between different layers, such as β-chitin-CNO, CNO-Ni–P, and several boundaries inside Ni–P, generating heterojunction capacitance to response with EMW due to unliked dielectric properties [52]. The uneven distribution of positive and negative charges on the interface will generate spatial electric dipole moment and polarization relaxation, thus strengthening dielectric loss. On basic of PNM model, rational structural design and heterointerface engineering immensely optimize the area of heterogeneous interface through core strategies, containing porous skeleton, nanostructures and multilayer construction. Moreover, large amounts of defects, functional groups, and dangling bonds locate are located in β-chitin, CON or Ni–P, where these dipoles play as polarization centers for dipole polarization loss. Thirdly, countless cavities in the β-chitin aerogel framework not only facilitates the filling of air that would enhance the impedance matching, but also act as dihedral angles to lengthen the propagation way of EMW, as well as assisting the multiple reflection of inside EMW [53]. Fourth, Ni–P particles, as a ferromagnetic medium, offer magnetic response under the alternating EM fields. Magnetic loss, including resonance and eddy current loss, is offered by magnetic Ni–P, which perform as a ferromagnetic medium to induce magnetic response under the alternating EM fields [54]. Evidently, fluctuated C_0_-f curve means that the natural resonance and exchange resonance happen simultaneously at high or low frequencies, accompanying with eddy current loss in whole measured frequency range (Fig. S10) [55]. And the moderate electrical conductivity of the outermost Ni–P layer also helps easing the reflection of EMW and thus promoting the absorption to a great extent. Finally, it's worth noting that the conductive loss, MWSE and magnetic loss can be controlled by adjusting the rounds of electroless plating. To be specific, when the rounds of electroless plating close to zero, the conductivity loss s plays a dominant role as a result of the few Ni–P coating on the surface of CNO layer. As the number of rounds increased, the MWSE and magnetic loss improved dramatically due to the strengthening of Ni–P-CNO interfaces, which in formed a sharp contrast with the degressive conductivity, thus leading to a shift in dominance. The whole process seems like the story of “tortoise and the Hare” in Aesop's fable, where the tortoises are magnetic loss and MWSE while the hare is conductive loss.

### EMI Shielding Performance of CONF

**Fig. 8:**
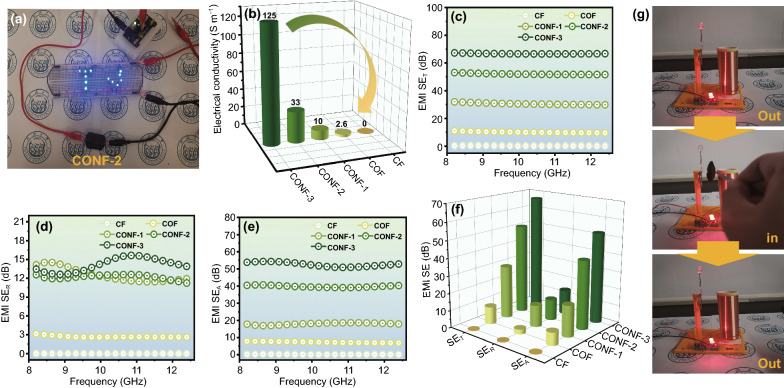
**a** Digital image of the CONF-2 that light the “TJ” LED lamps; electrical conductivity **b**, SE_T_ **c**, SE_A_ **d**, and SE_R_ **e** in X-band of as-prepared films; **f** comparison of average SE_T_, SE_A_, and SE_R_; **g** the digital images of a facial wireless power transmission device with CONA-2

Figure 8 displays the conductivity (*σ*) and EMI shielding performances of the CONF, which is also prepared with β-chitin, CNO and Ni–P as constitutional component. In addition, the specific thickness of each film is given in Table S2. The luminous LED lights in digital image indicate the low resistivity of CONF-2 (Fig. 8a). For Fig. 8b, the σ of the pure CF is close to 0, and the slightly improved σ (2.6 S m^−1^) of COF is almost unchanged compared with the pure CF. After the treatment of electroless plating, the films covered with crescent Ni–P exhibit rapidly decreasing resistivity, and the σ of CONF-1, CONF-2, and CONF-3 progressively grow to 10, 33, and 125 S m^−1^, respectively. In view of close correlation between σ and EMI shielding performance, the total shielding effectiveness (SE_T_) in X-band inherits a similar tendency with σ, which is also explained with Simon's formula [56]. From Fig. 8c, it is easily found that the pure CF basically fails in obtaining EMI shielding performance. The combination of CNO slightly improves the SE_T_ value of COF (10.0 dB), but the finite elevation still meets the actual requirements unsuccessfully (at least 20 dB). For the electroless plating of amorphous Ni–P layer, the SE_T_ of CONF-1 significantly increases to 30 dB. With the rise in the round of electroless plating, the SE_T_ of the CONF-2 and CONF-3 sequentially increase to 51.9 and 66.6 dB, respectively, thus reaching the maximum 6 times of EMI shielding efficiency than COF. Generally speaking, EMI shielding performance is divided into the reflection (SE_R_) part and absorption (SE_A_) part, which corresponds to the charge migration, polarization relaxation, magnetic response and multiple reflection [57, 58]. As shown in Fig. 8d, a huge promotion of SE_R_ is discovered from COF to CONF-1, implying that the outermost layer of Ni–P enhances the reflection of the film to EMW, so it plays an important role in ameliorating the shielding performance. Besides, a similar reinforcement also occurs in SE_A_ because electromagnetic coupling network from magnetic Ni–P layer and conductive CNO layer also enhances EMW absorption. With the increase of electroless plating rounds, SE_R_ fluctuates almost at a similar value about 13 dB while SE_A_ keeps rising. According to average EMI shielding parameter in X-band, the SE_A_ is always higher than the SE_R_, suggesting an absorption dominant EMW shielding mechanism (Fig. 8f). Overall, as CNO is wrapped by β-chitin in the process of hot pressing, the continuous conductive network is limit to a certain extent, which is reflected in the low EMI shielding performance of COF. Under the circumstances, CNO with large specific surface areas induce boosted heterogeneous interface with β-chitin nearby, making great contribution to the MWSE and following EMW energy response. Afterward, magnetic component modified CONF further strengthen the MWSE via interaction between β-chitin and Ni–P particles, which is also essential for concomitant magnetic loss and intensifying reflection. In addition, according to the principle of Tesla coil, a homemade wireless power transmission system is set as a typical example for EMI shielding effect visually, where the tunable light/off LED proves efficient EMI shielding performance of CONF-2 in 50 Hz (Fig. 8g and Movie S1).

### Thermal Management of CONA and CONF

**Fig. 9:**
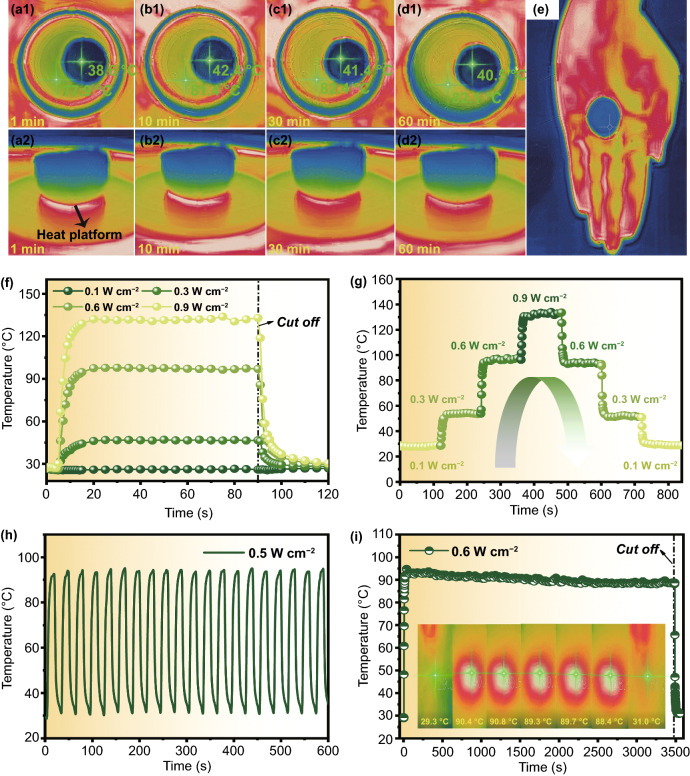
Top view **a1**-**d1** and side view **a2**-**d2** thermal infrared images of CONA-2 on 85 °C platform captured at 1 min/10 min/30 min/60 min, respectively; **e** thermal infrared images of CONA-2 placed on the hand. Photothermal property of the CONA-2: **f** photothermal property of the CONF-2 at different power densities; **g** photothermal property of CONF-2 under gradually changing power density; **h** photothermal stability of the CONF-2 in 20 light/unlight cycles; **i** long-term photothermal stability of the CONF-2 within 3600 s

Nowadays, the multifunction of EMW absorbers/shield is required to fulfill the ever-growing demands in complex environment and situation, and it is regarded as an advanced development direction of new-generation EMW materials [59]. Among them, thermal management, including joule heating, thermal conduction/insulation, photothermal and infrared stealth, attracts our eyes for its characteristic of energy storage and environmental protection [60–62]. Herein, the thermal insulation and photothermal property of CONA-2 and CONF-2 are visually investigated, respectively. Specifically speaking, CONA-2 is settled on the heating platform with setting temperature of 85 °C, and the practical temperature variation is calibrated in Fig. S1. For Fig. 9a1-d1, the top view thermal infrared images of the CONA-2 at 1, 10, 30, and 60 min are presented. As displayed in top view thermal infrared images (Fig. 9a1-d1), the surface temperature of CONA-2 still (thickness = 10 mm) remains unchanged with the increased time, and it fluctuates around 40 °C after being heated for 60 min, nearly 1/2 of the heating platform temperature, implying the satisfactory long-term thermal insulating stability of fabricated CONA-2. Additionally, side view thermal infrared images present that the temperature gradually decreases from the bottom in contact with the heating plate to the ambient temperature at half the thickness (Fig. 9a2-d2). The total thermal conductivity of samples depends on thermal conduction, thermal convention and thermal radiation in solid medium, gas medium, and radiative heat transfer, respectively. The low density and porous structure of the CONA-2 encounter an obstruction in thermal transport because air in the CONA-2 processes a lower thermal conductivity than the solid phase, and thus, CONA-2 can ensure its usage in high temperature circumstances, as well as potential infrared stealth function. As seen in Fig. 9e, different from the high infrared radiation intensity of the bare hand, the color of the hand with CONA-2 covered turns red (36.7 °C) to green (29.7 °C), making it invisible in infrared detection device. Furthermore, photothermal property of CONF-2 is studied under exposing to an 808 nm NIR laser with power densities from 0.1 to 0.9 W cm^−2^, and the measured sample (2.0 × 2.0 cm^2^) is put on the platform. The increase of saturated temperature of CONF-2 is not obvious at 0.1 W cm^−2^ in comparison of ambient temperature (Fig. 9f). Whereafter, its saturated temperature raises with the increased power densities, which reach high values of 46, 97, and 131 °C at 0.3, 0.6, and 0.9 W cm^−2^, respectively. It is noteworthy that CONF-2 can reach and leave its saturated temperatures in split second (within 15 s), suggesting excellent photothermal response efficiency. Figure 9g reveals the temperature-times curve under the one periodic stepwise from 0.1 to 0.9 W cm^−2^, and back to 0.1 W cm^−2^. It clear that a tunable and quick-response photothermal property of the CONF-2 is obtained. For further analyzing the reliability and long-time stability of CONF-2 as a photothermal equipment, a cyclic heating/cooling test (Fig. 9h) and long-term stability test (Fig. 9i) are employed with a power density of 0.6 W cm^−2^. The heating/cooling curve of 1th cycle and 20th cycle are almost same and the CONF-2 rises to its saturated temperature within 15 s and remains stable over 3400 s, suggesting remarkable photothermal durability. On the one hand, CNO processes the characteristics of *sp*^3^ and *sp*^2^ hybridization, π electron cloud, close energy level, wide band gap and broad light absorption range, which promotes the absorption of the photons in the light wave, thereby causing electrons to undergo energy transition. On the other hand, CNO can absorbs photon more thoroughly due to its blackness property, and thus converts photo-energy into heat energy efficiently. Besides, the EMI shielding performance of the CONF-2 after long-term photothermal stability test is measured in Fig. S12, where the CONF-2 still holds excellent EMI shielding performance.

## Conclusions

In summary, for the first time, a heterointerface engineering strategy is proposed for obtaining high-efficiency EMW absorbing/shielding materials with boosted MWSE. With the assistant of PNM model, the squid pen derived β-chitin/carbon nano-onions/Ni–P hierarchical aerogel was successfully fabricated by electrostatic assembly and electroless plating techniques. The heterogeneous interface area is optimized to the maximum extent due to the porous skeleton, nanostructure and multilayer construction on the basic of PNM model. By regulating the round of the electroless plating, the tunable EMW parameter and absorption performance are achieved, where the RL_min_ and EAB of CONA-2 at the thickness of 2.67 mm are − 50.83 dB and 6.8 GHz, respectively. The conductive loss and polarization/magnetic loss of CONA exhibit opposite trends, as describing like the story of “The Hare and the Tortoise”. With similar material composition and preparation methods, a flexible CONF was prepared and the EMI shielding in the X-band reach 66.66 dB at a thickness of 82 µm. In addition, the hierarchical CONA and CONF also possessed excellent thermal insulation and photothermal properties, respectively. Our findings provide a unique design perspective and inspiration for the construction of MWSE boosted EMW response materials, as well as the exploration of other multifunction to deal with harsh environment.

## Supplementary Information

Below is the link to the electronic supplementary material.

Supplementary file1 (PDF 1110 kb)

Supplementary file2 (MP4 1561 kb)
