# Supplementary material for: Heterointerface Engineering of β-Chitin/Carbon Nano-Onions/Ni–P Composites with Boosted Maxwell-Wagner-Sillars Effect for Highly Efficient Electromagnetic Wave Response and Thermal Management
Source: Nanomicro Lett. 2022 Mar 29;14:85. doi: 10.1007/s40820-022-00804-w (PMC8964898; doi:10.1007/s40820-022-00804-w)
Supplement: Supplementary file 1 — Supplementary file1 (PDF 1110 kb) [file 40820_2022_804_MOESM1_ESM.pdf]

Supporting Information for

Heterointerface Engineering of β-Chitin/Carbon Nano-Onions/Ni-P Composites with Boosted Maxwell-Wagner-Sillars Effect for Highly Efficient Electromagnetic Wave Response and Thermal Management

Fei Pan[1], Lei Cai[1], Yuyang Shi[1], Yanyan Dong[1], Xiaojie Zhu[1], Jie Cheng[1], Haojie Jiang[1], Xiao Wang[1], Yifeng Jiang[2], Wei Lu[1, *]

[1]Shanghai Key Lab. of D&A for Metal-Functional Materials, School of Materials Science & Engineering, Tongji University, Shanghai 201804, P. R. China

[2]School of Materials Science and Engineering, University of New South Wales, Sydney 2052, Australia

*Corresponding author. E-mail: weilu@tongji.edu.cn (Wei Lu)

S1 RCS Simulation

The RCS simulation of EM absorber was used CST software based on far-field response. In this simulation, the constructed model consists of the absorber/ paraffin layer and the perfect electric conductor (PEC) layer, where PEC also is also regarded as a reference value to determine the RCS reduction. In detail, the length and width of each layer were set as 200 mm and the thicknesses of the absorber/paraffin layer (35% filling ratio) and the PEC layer were set as 2.67 and 5.00 mm, respectively. The simulation used plane wave excitation, and the EMW propagates in the negative direction of the x-axis and the electric polarization direction is along the z-axis. In addition, the free space boundary conditions were used and the center frequency was defined as 11 GHz. The RCS value of the simulated FFSC uses the time domain method for calculation and the detail equation expressed as following:

$$\sigma(dB\, m^2) = 10log\left[\frac{4\pi S}{\lambda^2}\left|\frac{E_s}{E_i}\right|^2\right]$$

where S is the area of the layer, λ is the length of the incident EMW, E_s and E_i are the electric field intensity of transmitting waves and receiving waves, respectively.

Formula S1

calculated delta value

$$|\triangle| = \left|sinh^2(Kfd) - M\right|$$

$$K = \frac{4\pi\sqrt{\mu'\varepsilon'} \times sin\left(\frac{\delta_e + \delta_m}{2}\right)}{c \times cos\delta_e \times cos\delta_m}$$

$$M = \frac{4\mu' cos\delta_e \times \varepsilon' cos\delta_m}{(\mu' cos\delta_e - \varepsilon' cos\delta_m)^2 + \left[tan\left(\frac{\delta_{m-\delta_e}}{2}\right)\right]^2 \times (\mu' cos\delta_e + \varepsilon' cos\delta_m)^2}$$

S2 Formula of Shielding Performance

Films were cut into rectangular shapes with a size of 22.8 mm × 10.2 mm to match the waveguide device. The EMI shielding effectiveness is expressed as the total effectiveness (SET), the absorption effectiveness (SEA), and reflection effectiveness (SER):

$$SE_T = SE_A + SE_R + SE_M$$
$$R + A + T = 1$$
$$R = |S_{11}|^2 = |S_{22}|^2$$
$$T = |S_{12}|^2 = |S_{21}|^2$$
$$SE_R = 10log\left(\frac{1}{1-R}\right)$$
$$SE_A = 10log\left(\frac{1-R}{T}\right)$$

S3 Supplementary Figures and Tables

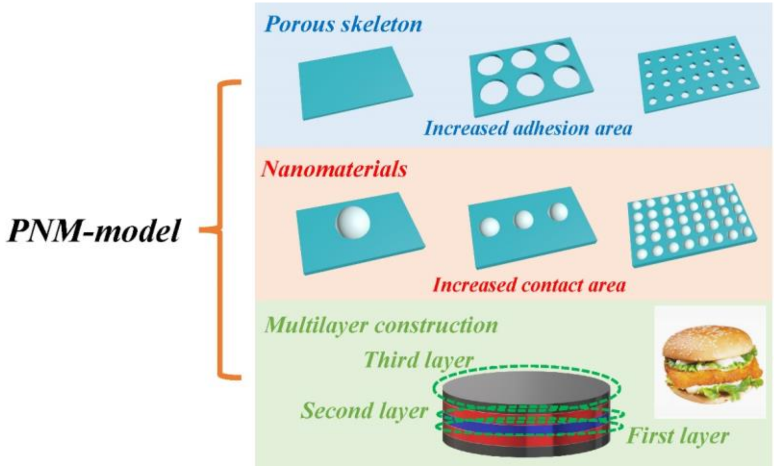

Fig. S1 Schematic illustrating PNM model in introduction

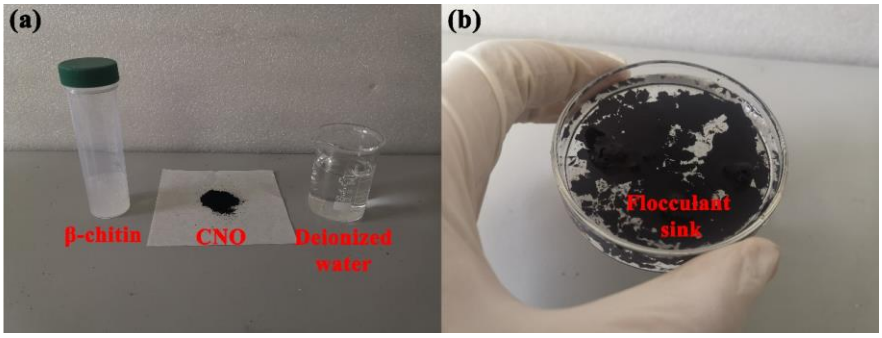

Fig. S2 Electrostatic adherence phenomenon between CNO and β-chitin

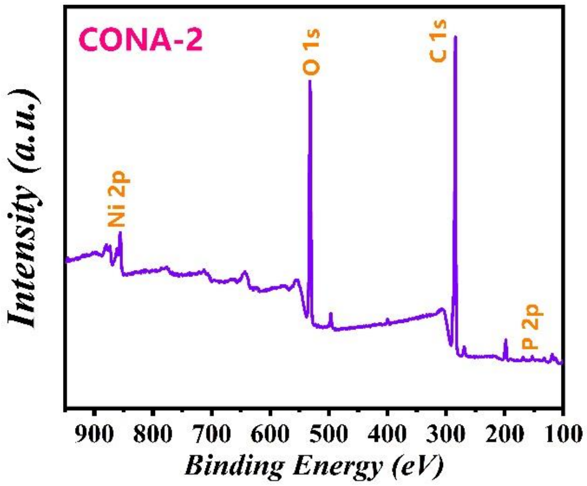

Fig. S3 Wide-scan XPS survey of CONA-2

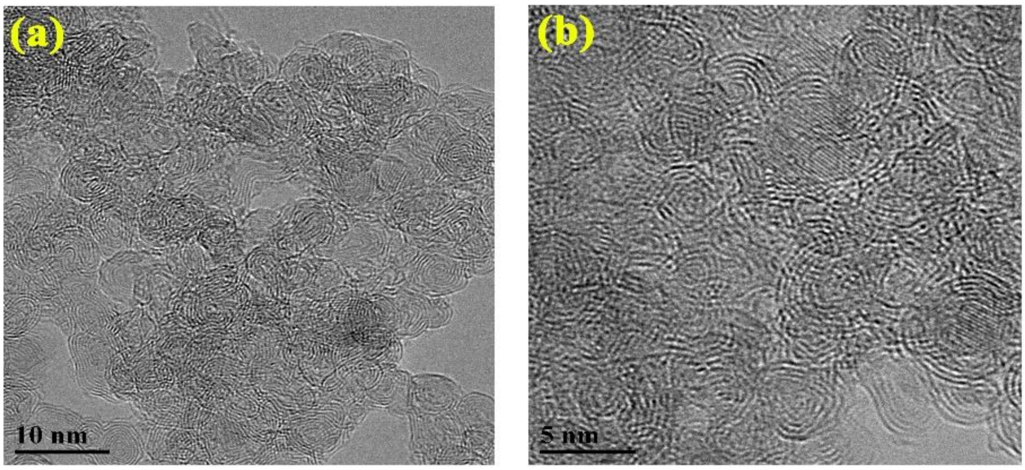

Fig. S4 TEM images of CNO

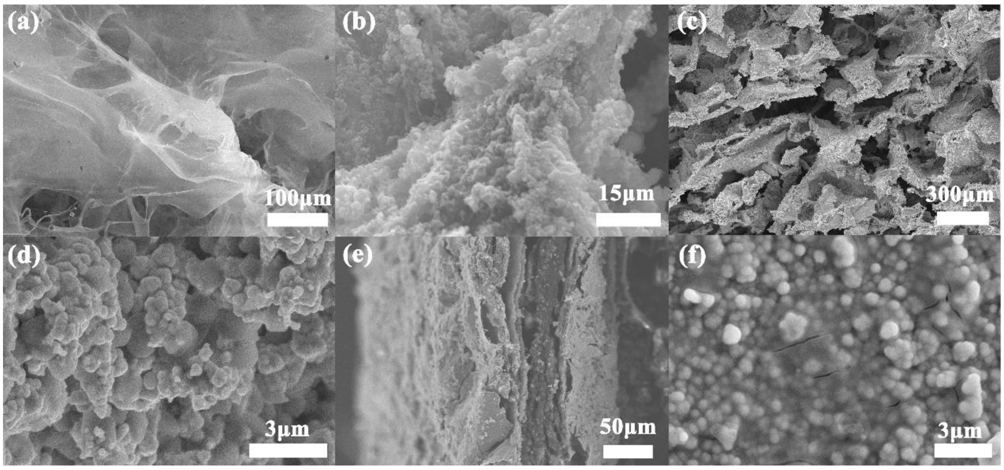

Fig. S5 SEM images of **a** CA, **b** COA, **c-d** CONA-2, and **e-f** CONF-2

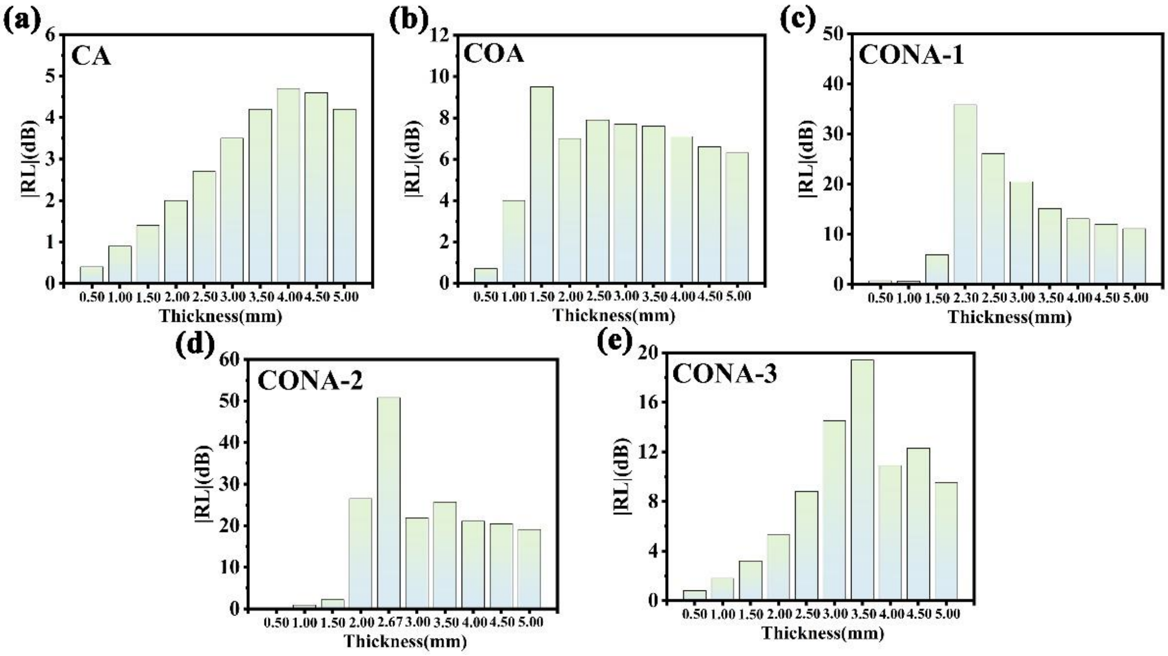

Fig. S6 Statistical RL value of **a** CA, **b** COA, **c** CONA-1, **d** CONA-2, and **e** CONA-2 with different thicknesses (1.0−5.0 mm)

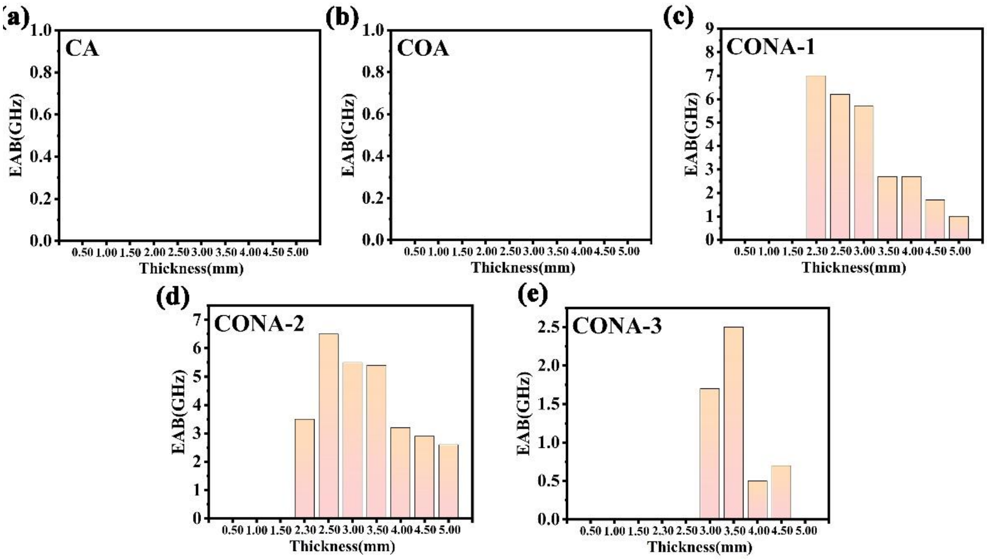

Fig. S7 Statistical EAB value of **a** CA, **b** COA, **c** CONA-1, **d** CONA-2, and **e** CONA-2 with different thicknesses (1.0−5.0 mm)

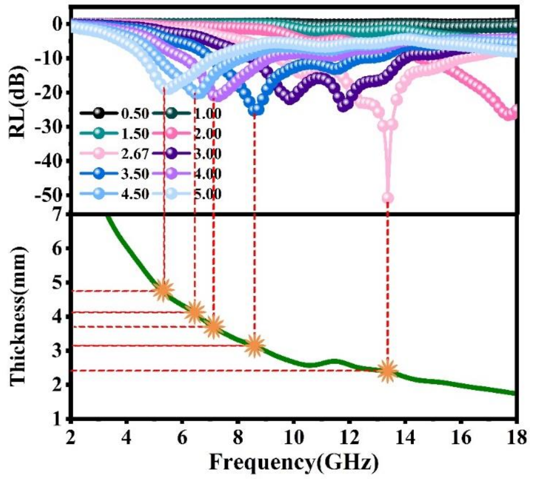

Fig. S8 Dependence matching thickness (t_m) on matching frequency of CONA-2

Table S1 Comparison of the specific EMW absorption performance in similar works

Sample	EAB(GHz)	thickness (mm)	RL	Refs.
C /MoS_2 aerogel	1.3	4.00	-43.00	[S1]
G/CNT/Fe_3O_4 aerogel	3.2	4.00	-49.00	[S2]
G/MXenes aerogel	4.2	4.40	-20.00	[S3]
N-Ni_xS_y/Co_xS_y@C aerogel	3.7	2.50	-47.20	[S4]
CNT/FeNi aerogel	4.2	2.00	-39.39	[S5]
G/SiC aerogel	4.7	3.00	-47.30	[S6]
SiC aerogels	4.0	2.00	-43.00	[S7]
G/polyethylene glycol aerogel	5.3	2.35	-43.20	[S8]
Polyaniline/G aerogel	3.2	3.00	-42.30	[S9]
$CoFe_2O_4$ /CNT/ polyurethane aerogel	2.3	6.00	-45.80	[S10]
CONA-2	6.8	2.67	-50.83	**this work**

C:Carbon G:Graphene

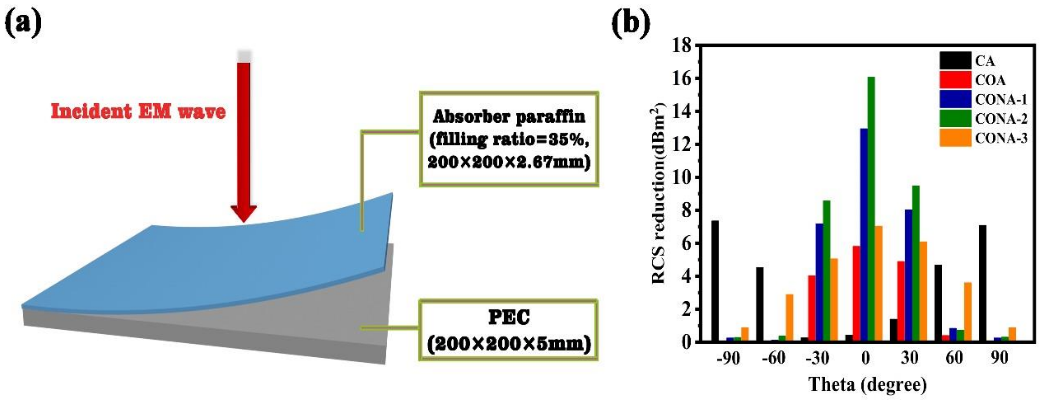

Fig. S9 RCS schematic diagram and RCS reduction achieved by subtracting the samples with the PEC

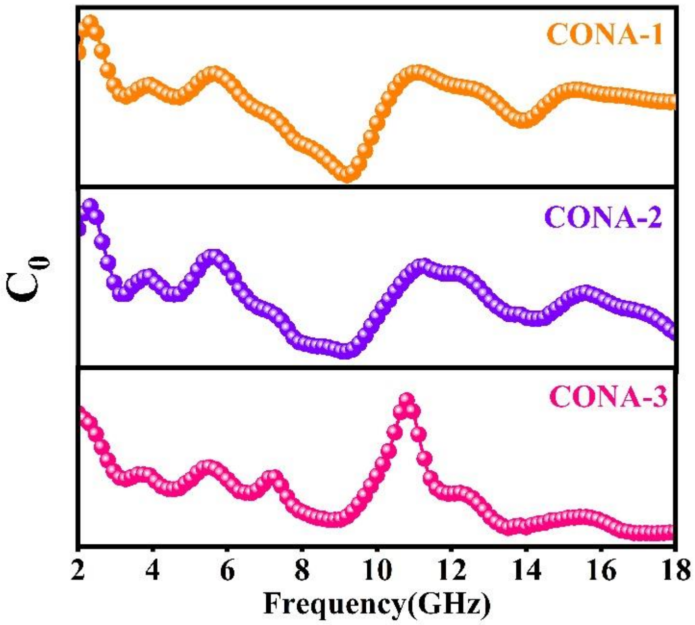

Fig. S10 C_o value of samples

Table S2 The thickness of all films in electromagnetic shielding measurement

Sample	thickness (μm)
CF	39
COF	41
CONF-1	63
CONF-2	82
CONF-3	130

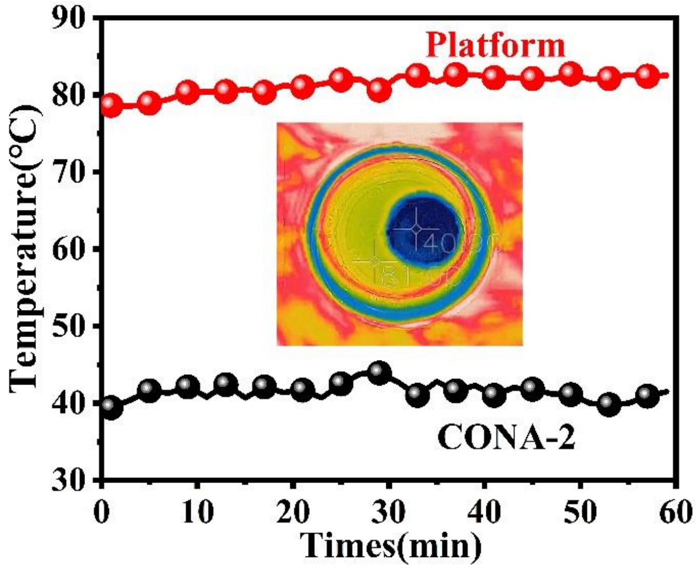

Fig. S11 Heating time versus sample temperature line charts of the CONA-2

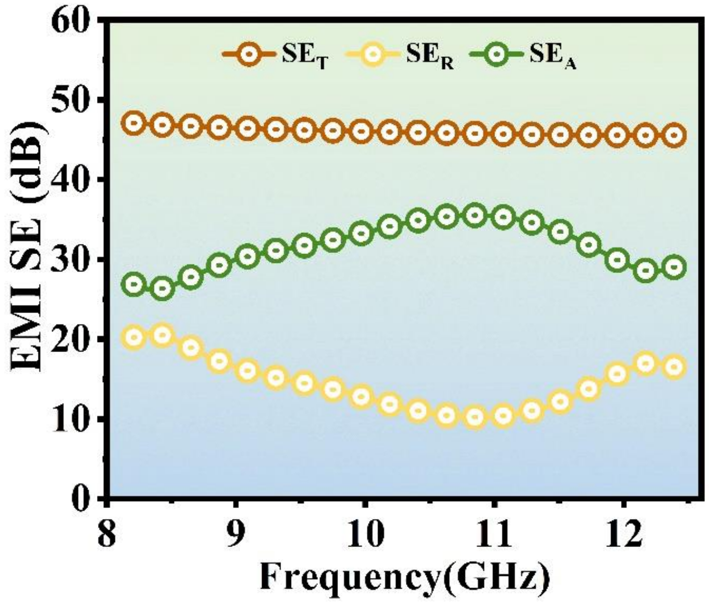

Fig. S12 EMI shielding performance of the CONF-2 in air after long-term photothermal stability test

Supplementary References

[S1] J.B. Cheng, H.B. Zhao, M. Cao, M.E. Li, A.N. Zhang et al., Banana leaflike C-doped MoS_2 aerogels toward excellent microwave absorption performance. ACS Appl. Mater. Interfaces **12**(23), 26301-26312 (2020). https://doi.org/10.1021/acsami.0c01841

[S2] Y. Qin, Y. Zhang, N. Qi, Q. Wang, X. Zhang et al., Preparation of graphene aerogel with high mechanical stability and microwave absorption ability via combining surface support of metallic-CNTs and interfacial cross-linking by magnetic nanoparticles. ACS Appl. Mater. Interfaces **11**(10) , 10409-10417 (2019). https://doi.org/10.1021/acsami.8b22382

[S3] X.L. Li, X.W. Yin, C.Q. Song, M.K. Han, H.L. Xu et al., Self-assembly core-shell graphene-bridged hollow MXenes spheres 3D foam with ultrahigh specific EM

absorption performance. Adv. Funct. Mater. **28**(41), 1803938 (2018). https://doi.org/10.1002/adfm.201803938

[S4] L. Gai, G. Song, Y. Li, W. Niu, L. Qin et al., Versatile bimetal sulfides nanoparticles-embedded N-doped hierarchical carbonaceous aerogels (N-Ni_xS_y/Co_xS_y@C) for excellent supercapacitors and microwave absorption. Carbon **179**, 111-124 (2021). https://doi.org/10.1016/j.carbon.2021.04.029

[S5] J. Xu, X. Zhang, H.R. Yuan, S. Zhang, C.L. Zhu et al., N-doped reduced graphene oxide aerogels containing pod-like N-doped carbon nanotubes and FeNi nanoparticles for electromagnetic wave absorption. Carbon **159**, 357-365 (2020). https://doi.org/10.1016/j.carbon.2019.12.020

[S6] Y. Jiang, Y. Chen, Y.J. Liu, G.X. Sui, Lightweight spongy bone-like graphene@SiC aerogel composites for high-performance microwave absorption. Chem. Eng. J. **337**, 522-531 (2018). https://doi.org/10.1016/j.cej.2017.12.131

[S7] C.Y. Liang, Z.J. Wang, Eggplant-derived SiC aerogels with high-performance electromagnetic wave absorption and thermal insulation properties. Chem. Eng. J. **373**, 598-605 (2019). https://doi.org/10.1016/j.cej.2019.05.076

[S8] H.M. Ji, J. Li, J.J. Zhang, Y. Yan, Remarkable microwave absorption performance of ultralight graphene-polyethylene glycol composite aerogels with a very low loading ratio of graphene. Compos. Part A Appl. Sci. Manuf. **123**, 158-169 (2019). https://doi.org/10.1016/j.compositesa.2019.05.012

[S9] Y. Wang, X. Gao, Y.Q. Fu, X.M. Wu, Q.G. Wang et al., Enhanced microwave absorption performances of polyaniline/graphene aerogel by covalent bonding. Compos. Part B Eng. **169**, 221-228 (2019). https://doi.org/10.1016/j.compositesb.2019.04.008

[S10] J. Luo, Y. Wang, Z. Qu, W. Wang, D. Yu, Lightweight and robust cobalt ferrite/carbon nanotubes/waterborne polyurethane hybrid aerogels for efficient microwave absorption and thermal insulation. J. Mater. Chem. C **9**(36), 12201-12212 (2021). https://doi.org/10.1039/D1TC02427B
